# Common arterial trunk and ventricular non-compaction in *Lrp2* knockout mice indicate a crucial role of LRP2 in cardiac development

**DOI:** 10.1242/dmm.022053

**Published:** 2016-04-01

**Authors:** Maria E. Baardman, Mathijs V. Zwier, Lambertus J. Wisse, Adriana C. Gittenberger-de Groot, Wilhelmina S. Kerstjens-Frederikse, Robert M. W. Hofstra, Angelika Jurdzinski, Beerend P. Hierck, Monique R. M. Jongbloed, Rolf M. F. Berger, Torsten Plösch, Marco C. DeRuiter

**Affiliations:** 1 Department of Genetics, University Medical Center Groningen, University of Groningen, Hanzeplein 1, Groningen 9713 GZ, The Netherlands; 2 Department of Pediatrics, University Medical Center Groningen, University of Groningen, Hanzeplein 1, Groningen 9713 GZ, The Netherlands; 3 Department of Anatomy and Embryology, Leiden University Medical Center, PO-Box 9600, Leiden 2300 RC, The Netherlands; 4 Department of Clinical Genetics, Erasmus Medical Center Rotterdam, PO-Box 2040, Rotterdam 3000 CA, The Netherlands; 5 Neural Development and Gastroenterology Units, UCL Institute of Child Health, London WC1 NEH, UK; 6 Department of Cardiology and Department of Anatomy and Embryology, Leiden University Medical Center, PO-Box 9600, Leiden 2300 RC, The Netherlands; 7 Center for Congenital Heart Diseases, Beatrix Children's Hospital, University Medical Center Groningen, University of Groningen, Hanzeplein 1, Groningen 9713 GZ, The Netherlands; 8 Department of Obstetrics and Gynaecology, University Medical Center Groningen, University of Groningen, Hanzeplein 1, Groningen 9713 GZ, The Netherlands

**Keywords:** Cardiac outflow tract, Heart development, Lipoprotein-related receptor protein 2, Neural crest, Second heart field

## Abstract

Lipoprotein-related receptor protein 2 (LRP2) is important for development of the embryonic neural crest and brain in both mice and humans. Although a role in cardiovascular development can be expected, the hearts of *Lrp2* knockout (KO) mice have not yet been investigated. We studied the cardiovascular development of *Lrp2* KO mice between embryonic day 10.5 (E10.5) and E15.5, applying morphometry and immunohistochemistry, using antibodies against Tfap2α (neural crest cells), Nkx2.5 (second heart field), WT1 (epicardium derived cells), tropomyosin (myocardium) and LRP2. The *Lrp2* KO mice display a range of severe cardiovascular abnormalities, including aortic arch anomalies, common arterial trunk (persistent truncus arteriosus) with coronary artery anomalies, ventricular septal defects, overriding of the tricuspid valve and marked thinning of the ventricular myocardium. Both the neural crest cells and second heart field, which are essential for the lengthening and growth of the right ventricular outflow tract, are abnormally positioned in the *Lrp2* KO. This explains the absence of the aorto-pulmonary septum, which leads to common arterial trunk and ventricular septal defects. Severe blebbing of the epicardial cells covering the ventricles is seen. Epithelial-mesenchymal transition does occur; however, there are fewer WT1-positive epicardium-derived cells in the ventricular wall as compared to normal, coinciding with the myocardial thinning and deep intertrabecular spaces. LRP2 plays a crucial role in cardiovascular development in mice. This corroborates findings of cardiac anomalies in humans with *LRP2* mutations. Future studies should reveal the underlying signaling mechanisms in which LRP2 is involved during cardiogenesis.

## Abstract (precis)

**Summary:** This paper sheds a new light on the role of the second heart field and neural crest cells in outflow tract formation in the mouse embryo. Depletion of the LPR2 results in a disturbed contribution pattern and subsequent common arterial trunk.

## INTRODUCTION

Lipoprotein-related receptor protein 2 (LRP2), also known as megalin, is a transmembrane glycoprotein receptor on the surface of epithelial cells, and belongs to the low-density-lipoprotein receptor (LDLR) family. The gene is mapped to chromosome 2 in the mouse as well as in human (Korenberg et al., 1994). LRP2 is highly expressed throughout development, starting in the eight-cell stage, and is limited to the trophectoderm in the blastocyst stage (Assemat et al., 2005). After implantation, it is expressed on the maternal-fetal interface, including in the trophectoderm, the visceral endoderm of the yolk sac and the placenta (Gueth-Hallonet et al., 1994). During further development, LRP2 is detected in many epithelial tissues, including the neuroepithelium, the kidney proximal tubule epithelial cells and the pericardium (Assemat et al., 2005). LRP2 is a multi-ligand receptor and capable of binding important signaling molecules, such as bone morphogenetic protein 4 (BMP4) (Spoelgen et al., 2005), sonic hedgehog (Shh) (McCarthy and Argraves, 2003), retinol binding protein (RBP) (Christensen et al., 1999) and vitamin B12 (Moestrup et al., 1996). Furthermore, LRP2 is a low-density lipoprotein (LDL) receptor on the yolk sac and placenta, and is involved in cholesterol transport as a co-transporter with cubilin (Christensen and Verroust, 2002).

Targeted disruption of the gene in mice results in severely malformed offspring with holoprosencephaly, neural tube defects, limb anomalies and a 100% lethality *in utero* or within 24 h after birth (Willnow et al., 1996). Disruption in humans is known as the autosomal-recessive Donnai-Barrow syndrome. Individuals with Donnai-Barrow syndrome display severe congenital malformations, including agenesis of the corpus callosum, sensorineural deafness, diaphragmatic hernia, omphalocele and nephritis (Kantarci et al., 2007). Literature reports of a few cases with heart anomalies such as double-outlet right ventricle, persistence of the left caval vein, persistent ductus arteriosus and ventricular septal defects; however, authors assume that because of early fetal loss of severe cases and limited examination of fetuses, the real incidence of heart anomalies could be much higher (Pober et al., 2009).

Interestingly, the effect of depletion of LRP2 on the cardiovascular development has never been described in mice, although expression of LRP2 on the mesothelial cells of the pericardial cavity has been reported together with expression of LRP2 in the neural crest (Assemat et al., 2005; Fisher and Howie, 2006). Previous studies have consistently shown that the epicardium, which arises from the pericardial mesothelium, and neural crest play important roles in the development of different cardiac structures such as the ventricular myocardium, the atrioventricular valves, the coronary arteries and the distal part of the outflow tract (Gittenberger-de Groot et al., 2000, 2012; Poelmann et al., 1998; Waldo et al., 2005; Wessels et al., 2012).

We examined in a developmental series the hearts of *Lrp2* knockout mouse embryos to elucidate a role of LRP2 in cardiac development and to determine which cardiac and/or extracardiac cell population is affected by the absence of the protein. We postulate that LRP2 plays a role in the formation of the compact ventricular myocardium through interaction with epicardium-derived cells (EPDCs). The transcription factor Wilms tumor 1 (WT1) is expressed in nuclei of the epicardium and in EPDCs during their early migration, whereas myocardial cells are negative for this protein (Moore et al., 1999), and is therefore considered to be a valid EPDC marker. We further examined the distribution patterns of cells from the neural crest (NCCs) (Jain et al., 2011; Keyte and Hutson, 2012; Poelmann et al., 1998; Waldo et al., 2005) and the second heart field (SHF) (Dyer and Kirby, 2009; Mjaatvedt et al., 2001; Scherptong et al., 2012; Waldo et al., 2001) because both are known to be crucial for outflow tract (OFT) septation and remodeling. Because LRP2 acts as a receptor in the retinoic acid (RA) and Shh signaling pathways, which are indispensable in SHF and NCC differentiation, we postulate a role for LRP2 in cardiac OFT formation. A valid and useful marker is Nkx2.5, which is highly expressed by cells from the SHF during their migration towards the OFT (Anderson et al., 2012; Scherptong et al., 2012). Extracardiac-positioned SHF cells that are Nkx2.5-positive, but negative for myocardial markers such as tropomyosin and myosin light chain kinase, migrate towards the distal OFT to establish right OFT lengthening, rotation and septation (Anderson et al., 2012). Failure of this process can result in OFT abnormalities (Bajolle et al., 2006). The contribution of NCCs to the OFT in *Lrp2* knockout and wild-type mice was studied by using Tfap2α, a transcription factor that is highly expressed by migrating and differentiating NCCs and not by cells of the SHF (Jain et al., 2011).

## RESULTS

### LRP2 is crucial for development of the cardiac outflow tract and atrioventricular canal

**Fig. 1.**
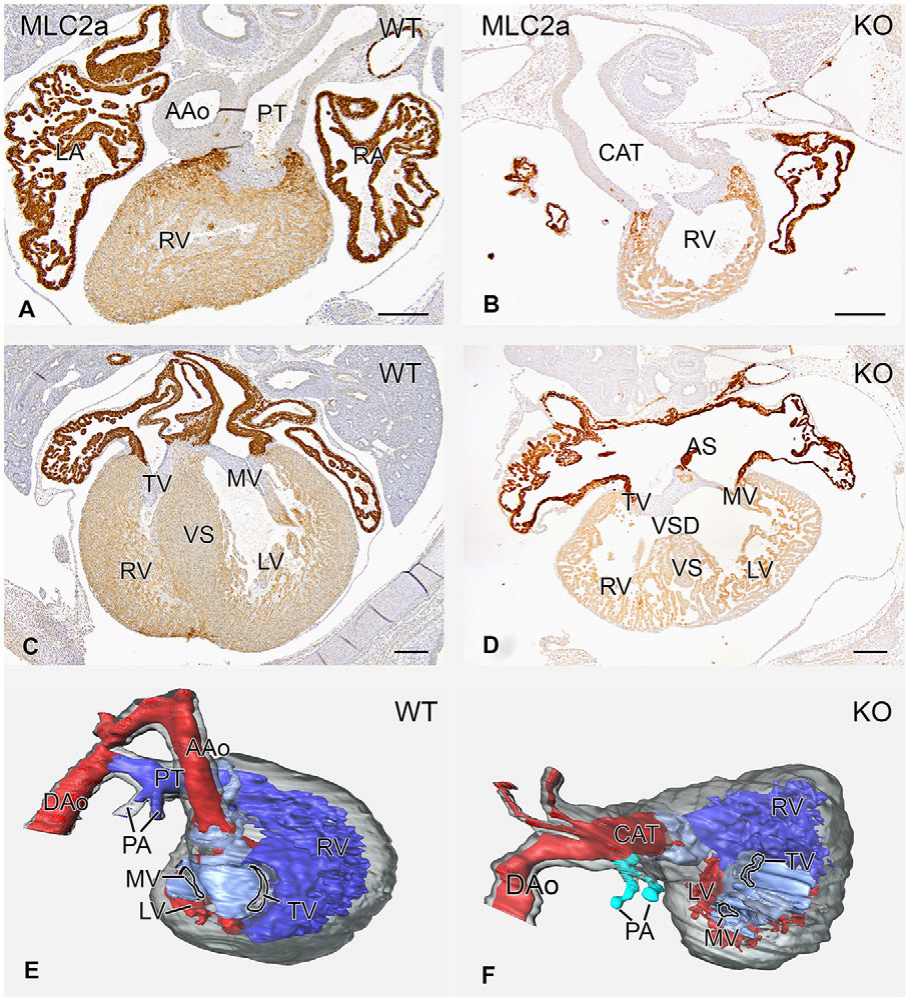
**Hearts of wild-type and knockout *Lrp2* mice at E15.5 stained for the myocardial marker MLC2A, and 3D reconstructions.** In wild-type (WT) embryos, both the ascending aorta (AAo) and pulmonary trunk (PT) are present (A,E). In the knockout (KO) embryos there is only one outflow vessel, a common arterial trunk (CAT) above the right ventricle (RV) (B,F). The atrioventricular canal (C,D) is normally marked by a tricuspid valve (TV) above the right ventricle (RV) and a mitral valve (MV) above the left ventricle (LV) with an intact ventricular septum (VS) (C). In the knockout embryo, the atrioventricular valves are present. The TV, however, overrides the inlet ventricular septal defect (VSD) and is positioned for its major part above the LV (D). There is a mild malalignment between the atrial septum (AS) and the VS. DAo, descending aorta; LA, left atrium; RA, right atrium. Scale bars: 200 µm.

Cardiac malformations were seen in all 17 analyzed *Lrp2* knockout embryos from E12.5 to E15.5 and are summarized in Table 1. The most obvious cardiac anomaly was a common arterial trunk (CAT; Fig. 1A,B,E,F) in 15/17 embryos. Eleven embryos with a CAT presented with a common pulmonary artery originating from the dorsal wall of the CAT before dividing into a right and left pulmonary artery. One embryo presented a double-outlet right ventricle (DORV). Ventricular inflow abnormalities were frequently observed, ranging from an overriding tricuspid orifice (*n*=7; compare Fig. 1C with 1D) to mitral valve atresia (*n*=1) and tricuspid valve stenosis (*n*=2). Aortic arch anomalies were seen in 4/9 knockout embryos at E15.5. Two embryos showed a right aortic arch with a retro-esophageal subclavian artery. Remarkably, at E15.5, all the embryos presented with abnormal coronary arteries. In 3/5 embryos we found a single left coronary artery, in 1/5 a single right coronary artery and in 1/5 a very wide left coronary artery in combination with a hypoplastic right coronary artery. There was no abnormally high take-off of the arteries seen (data not shown in figure).

**Table 1.**
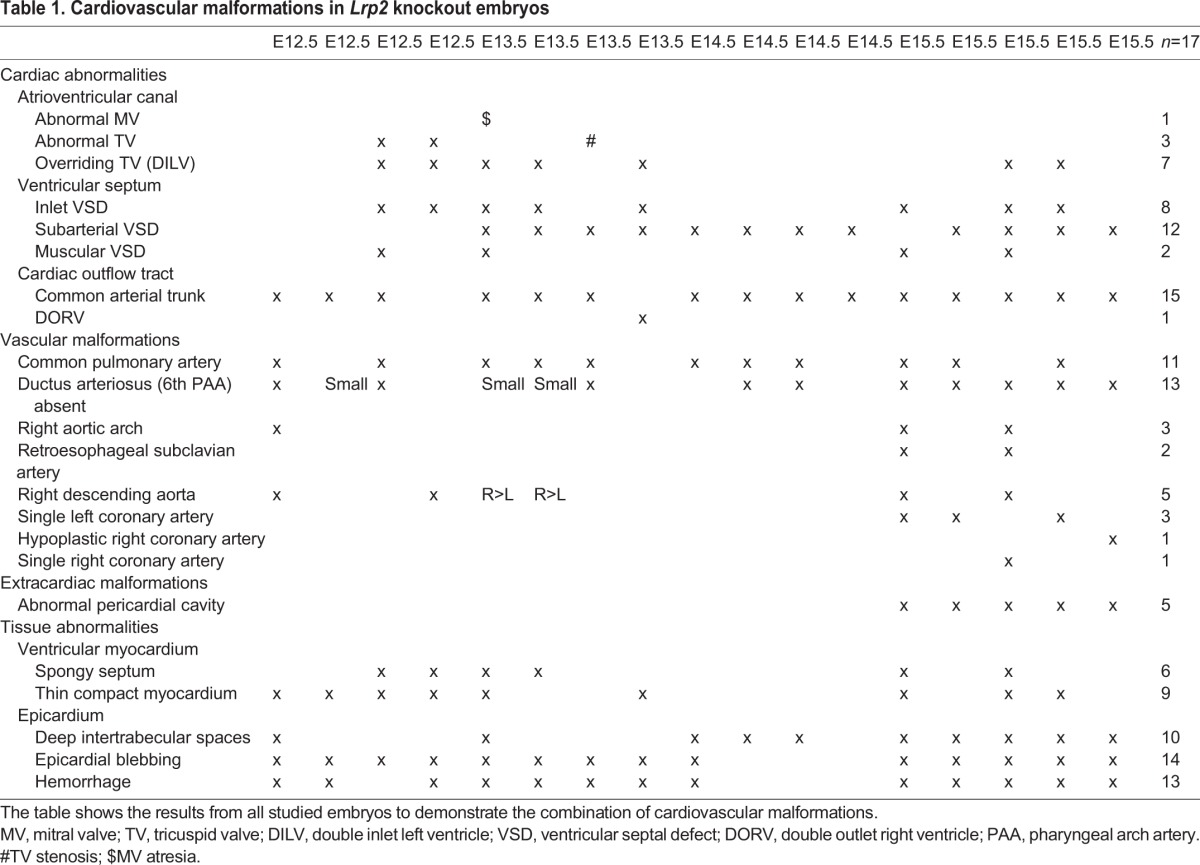
**Cardiovascular malformations in *Lrp2* knockout embryos**

Also at E15.5, in 5/5 embryos the right pleural cavity was not separated from the pericardial cavity, resulting in an abnormal position of the right lung next to the heart.

### LRP2 is crucial for migration and development of the second heart field and neural crest cells

**Fig. 2.**
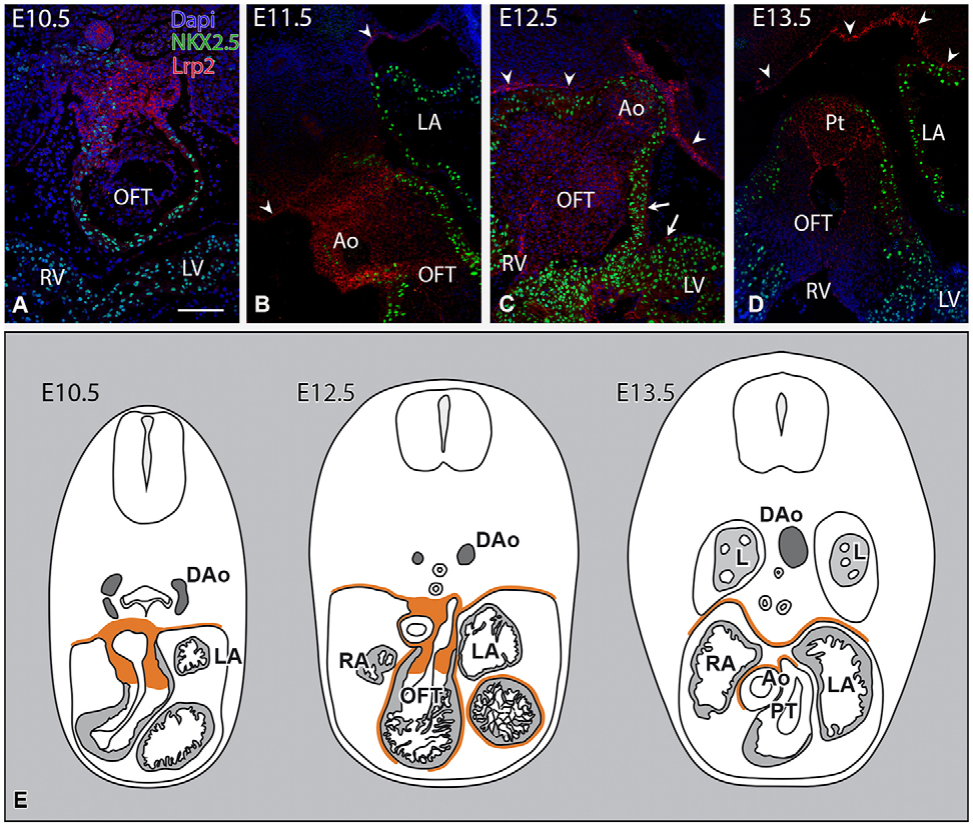
**LRP2 expression in the developing and septating cardiac OFT in combination with Nkx2.5 in wild-type mice at stage E10.5-E13.5, and schematic representation.** (A,B) LRP2 is expressed in the non-myocardial outflow tract (OFT) region, which contains neural crest and second heart field cells. (B,C) During septation from E11.5 to E12.5, the wall of the ascending aorta (Ao) and the pulmonary trunk (Pt) are LRP2-positive. (C,D) From E12.5, LRP2 expression becomes restricted to the endocardial and mesenchymal cells in the OFT cushions, with the marked mesenchymal prongs in the OFT septum. (D) In E13.5, the endocardial cells in the OFT become strongly LRP2-positive. Positive LRP2 cells are also seen in epicardium (arrows in C) and mesothelial lining of the pericardium (arrowheads). Scale bar: 100 µm. (E) Schematic representation. DAo, descending aorta; L, lung; LA, left atrium; LV, left ventricle; RA, right atrium; RV, right ventricle.

At E10.5, LRP2 was highly expressed in the mesenchyme surrounding the non-septated cardiac OFT region (Fig. 2A). The LRP2 expression was mainly in the non-myocardial cells (Nkx2.5-negative cells). During OFT septation, the wall of the ascending aorta (Fig. 2B) and pulmonary trunk remained LRP2-positive. The differentiating myocardium (Nkx2.5-positive) and epicardium were LRP2-negative at E11.5. At E12.5, LRP2 in the heart was mainly expressed by the mesenchymal cells in the OFT cushions (Fig. 2C), although the endocardial cells and the epicardium were also slightly positive. At E13.5, the LRP2 expression became more distinct in the developing intercalated cushions (Fig. 2D) and the expression in the epicardium was no longer detectable. In addition to the heart, the mesothelial lining of the pericardial cavity also expressed LRP2 from E10.5 to E13.5 (Fig. 2B-E).

**Fig. 3.**
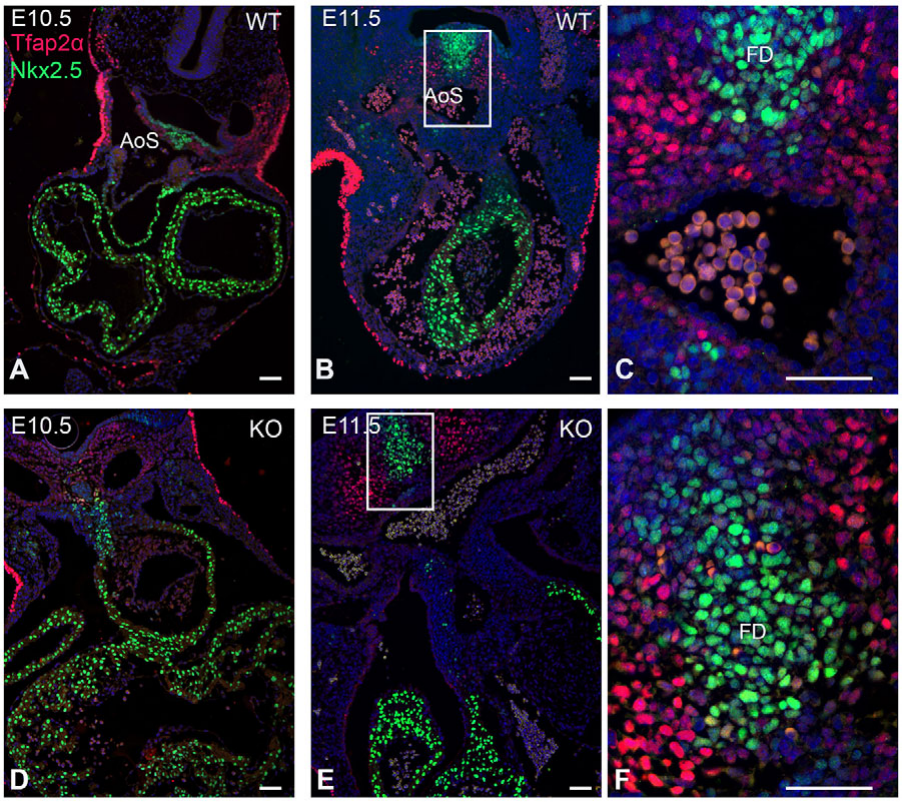
**Immunofluorescent Tfap2α and Nkx2.5 expression in the OFT area of the heart in wild-type and *Lrp2* knockout embryos at E10.5 and E11.5.** The mesenchymal Tfap2α (red)- and Nkx2.5 (green)-positive cells are separate populations in both the wild-type (WT) and knockout (KO) embryos. No co-expression was detected around the aortic sac (AoS) or the pharyngeal arch arteries. Note: a part of the ectodermal cells express Tfap2α, but are not involved in outflow tract (OFT) septation. The flow divider (FD) is a part of the Nkx2.5-positive cell population and is present in between the most caudal pharyngeal arch arteries (C,F). Boxed areas in B and E are shown in C and F, respectively. Scale bars: 50 µm.

In the non-myocardial OFT area (tropomyosin-negative), from which the aorta and pulmonary trunk develop, both NCCs (Tfap2α-positive) and SHF cells (Nkx2.5-positive) were found to be present (Fig. 3). Tfap2α and Nkx2.5 are not co-expressed in the stages studied. Therefore, these markers can be used to study the two separate cell populations. The non-myocardial Nkx2.5-positive mesenchymal cells are considered as SHF cells and will contribute to part of the smooth-muscle cell population of the ascending aorta and pulmonary trunk, as well as the myocardium of the OFT of both the left and right ventricle (Scherptong et al., 2012). Also, NCCs are present within this area and will contribute to the smooth-muscle cell population and OFT septum, but not to the myocardium of the OFT.

**Fig. 4.**
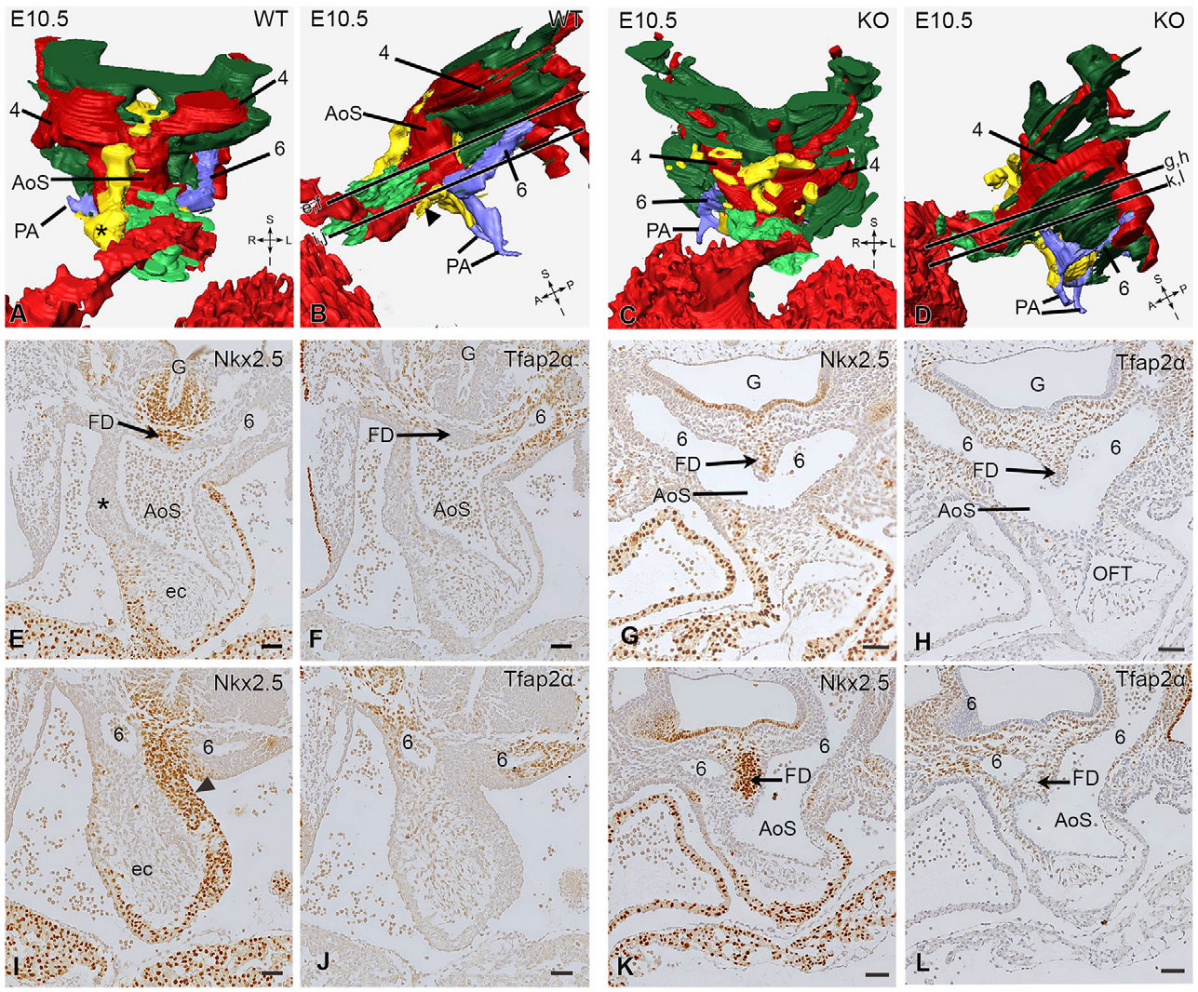
**Outflow tract septation characteristics with 3D reconstructions and histological sections of E10.5 wild-type and knockout embryos.** (A-D) In the reconstructions (WT, wild type; KO, knock out), the second heart field (SHF) has been depicted in yellow; the neural crest cells (NCCs) surrounding the aortic sac (AoS; in red) and the 4th (4; in red) and 6th (6; in blue) pharyngeal arch arteries have been removed. Only the NCC mesenchymal population (dark green) and the NCCs (bright green) that are embedded in the endocardial cushions (not shown) are depicted. The 6th pharyngeal arch arteries and the pulmonary arteries (PA) are depicted in blue. (A,E) Ventral view of a WT embryo: a population of SHF cells is seen to cross from dorsal to ventral, along the 4th pharyngeal arch artery on the right (aortic) side of the AoS up to the myocardial borderline of the AoS (asterisk). (B,I) Left lateral view. On the left (pulmonary) side of the AoS below the 6th pharyngeal arch artery, an SHF population is seen (black arrowhead). The section levels are indicated in B (upper section level: E,F and lower level: I,J). Not visible in the reconstructions is the Nkx2.5-positive SHF population in front of the gut (‘G’) that forms a ridge (FD, flow divider) in the dorsal wall of the AoS between the right and left 6th pharyngeal arch arteries (arrow in E) and negative for the NCC marker Tfap2α (arrow in F). NCCs are, however, abundantly present around the 6th pharyngeal arch arteries (brown Tfap2α cells in F,J). In the 3D reconstructions of the knockout embryo (C,D), the frontal SHF population is less well developed (C) and the population on the left (pulmonary) side remains in the midline dorsal position between the 6th pharyngeal arch arteries and does not properly extend anteriorly (D). The mesenchymal NCC population (dark green) on the left (pulmonary) side already reaches the NCCs in the endocardial OFT (D). The midline dorsal FD (arrows in G,H,K,L) protrudes prominently into the AoS and separates the 6th pharyngeal arch arteries. Scale bars: 50 µm. See also the online supplemental videos (E10.5_video_1080p.mpg) and interactive pdf reconstructions (E10.5_WT_revise.pdf and E10.5_KO_revise.pdf) at http://www.caskanatomy.info/research/supplement_megalin.html. S, superior; R, right; L, left; I, inferior; A, anterior; P, posterior; ec, endocardial cushions.

Already at E10.5 we found differences in the patterning and distribution of the Nkx2.5-positive SHF as well as the Tfap2α NCC population in the knockout population as compared to the control embryos (Fig. 4A-L). The SHF population in the wild-type embryos showed a mid-sagittal position in front of the aortic sac and provided a strand of cells between the 3rd and 4th pharyngeal arch artery towards the right (future aortic side) of the aortic sac (Fig. 4A). This population ended in the deep part of the (saddle-shaped) orifice level at the border of the myocardial OFT (Fig. 4A, asterisk). A second population of SHF cells was positioned immediately in front of the foregut, forming a flow divider (FD) in between the 6th pharyngeal arch arteries (Figs 3C and 4E, arrow). Remarkably, this FD was negative for NCCs, which were prominently present around the 6th pharyngeal arch arteries (Fig. 4F,J). The main body of the dorsal mid-sagittal SHF population was positioned in between the left and right 4th and the left and right 6th pharyngeal arch arteries, where it also showed a marked extension to the left side (pulmonary side) of the OFT (Fig. 4B,I, black arrowhead).

**Fig. 5.**
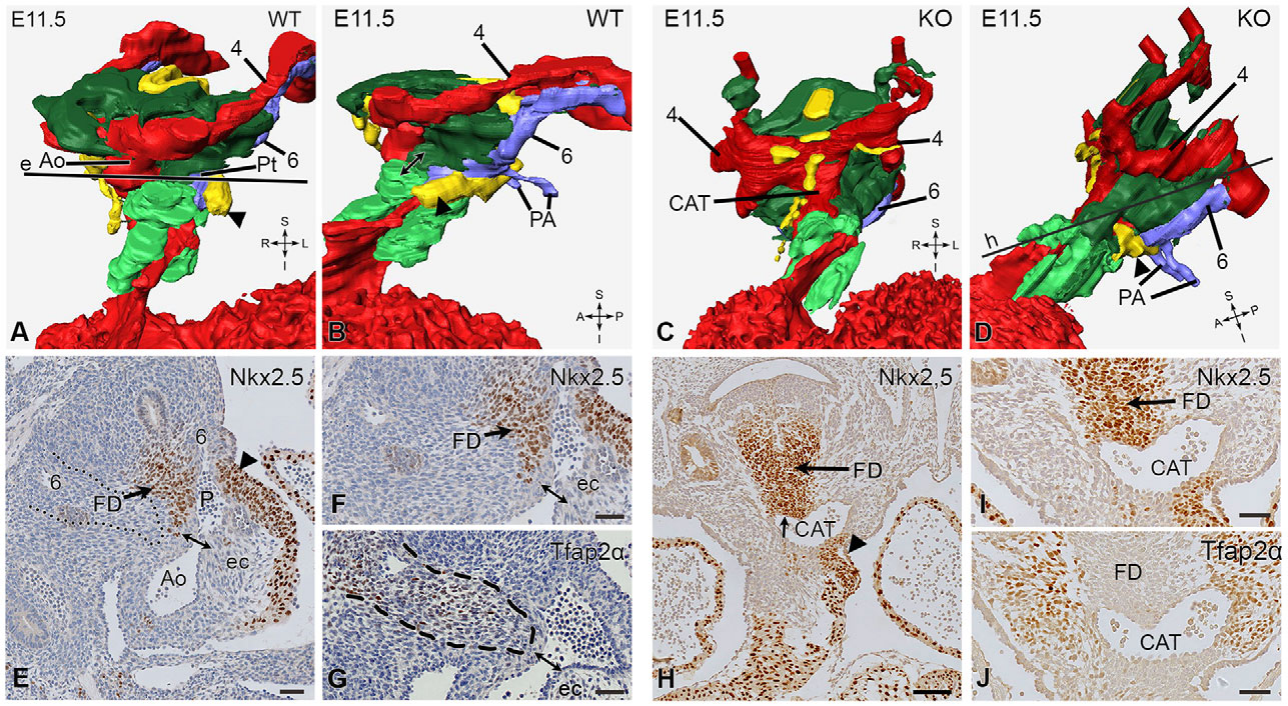
**Outflow tract septation characteristics with 3D reconstructions and histological sections of E11.5 wild-type and knockout embryos.** Comparable to Fig. 4, in the reconstructions (A-D) the second heart field (SHF) has been depicted in yellow; the neural crest cells (NCCs) surrounding the aorta (Ao; in red) and the 4th (4; in red) and 6th (6; in blue) pharyngeal arch arteries have been removed. Only the NCC mesenchymal population (dark green) and the NCCs (bright green) that are embedded in the endocardial cushions (not shown) are depicted. The 6th pharyngeal arch arteries and the pulmonary arteries (PA) are depicted in blue. The dorsal mid-sagittal SHF population has moved to the left and has extended a large band of cells to the left (pulmonary) side (P) of the OFT extending along the pulmonary vessel wall and the myocardial OFT (A,B,E, black arrowhead, F). The flow divider (FD) has separated the 6th pharyngeal arch arteries and has also moved to the left (E,F). The Tfap2α-positive NCCs (G) are encircled with a striped line. They have taken up a right-sided position next to the FD and are connected to the NCCs in the opposing endocardial cushions (ec). The NCC is forming the aorto-pulmonary septum (E-G, double-headed arrow), viewed in the 3D reconstruction (B) at the connection between dark and bright green. In the knockout embryo, both the SHF and the NCC population on the right (aortic) side are not well developed (C). Because the dorsal SHF population (not shown in the reconstruction) remains in midline (Nkx2.5-positive cells in sections H,I), there is hardly an extension along the left (pulmonary) side (C,D and H,I, black arrowhead). There is a marked Tfap2α (J) NCC population, however, on the left (pulmonary) side that connects to the bright green NCCs in the endocardial cushions. This does not lead to septation of the aortic sac, which now has become a common arterial trunk (CAT) (H-J). The FD is still prominently present and is Nkx2.5 (SHF)-positive (H,I, arrow) and Tfap2α (NCC)-negative (J; arrow). Section levels have been indicated in the reconstructions (A,C). Scale bars: 50 µm. See also the online supplemental videos (E11.5_video_1080p.mpg) and interactive pdf reconstructions (E11.5_WT_revise.pdf and E11.5_KO_revise.pdf) on http://www.caskanatomy.info/research/supplement_megalin.html. S, superior; R, right; L, left; I, inferior; A, anterior; P, posterior; Pt, pulmonary trunk.

At E11.5 this stream of SHF cells coursing between the 4th and 6th pharyngeal arch arteries became even more prominent (Fig. 5A,B,E,F). Together with a leftward and upward shift of the dorsal SHF population, it mainly contributed to the pulmonary OFT. This process has been referred to as ‘pulmonary push’ (Scherptong et al., 2012). During this leftward shift and extension of the SHF, also the FD that separates the left and right 6th pharyngeal arch arteries moves to the left (Fig. 5E,F, arrow). At this stage the NCCs were abundantly present around the pharyngeal arch arteries and extend with prongs into the endocardial cushions (Fig. 5A,B, bright green, and G). They formed the aorto-pulmonary septum separating the aortic sac into the aorta and the pulmonary trunk (Fig. 5E-G, double arrow). The pulmonary trunk was still connected to the 6th pharyngeal arch system and the connecting pulmonary arteries (Fig. 5B).

In the mutant embryos at E10.5 and E11.5, several differences were observed both in the distribution of the SHF and the NCC. The anteriorly located SHF population directed towards the right (aortic side) of the OFT was less well developed and less organized in the mutant embryo compared to the wild type (compare Fig. 4C to 4A). The main difference correlating with the development of a CAT was the diminished development of the left-sided SHF population that leads to the pulmonary push (compare Fig. 4D to 4B and 5D to 5B). The mid-sagittal SHF population remained in the mutant in the low position and did not move to the left (compare Fig. 5C,D to 5A,B, arrowhead). The NCCs were still abundantly present but in knockout embryos did not take a course that separates the aortic sac but followed a leftward (pulmonary side) swing to enter the endocardial cushions (Fig. 5D). This seems to be possible because of the lack of intervening SHF in this position (compare Fig. 5B to 5D). There was no septation of the aortic sac, resulting in a CAT with a FD in the dorsal position containing SHF cells but without NCCs (Fig. 5J) and thus connecting the 6th pharyngeal arch artery system and the pulmonary arteries to the CAT (Fig. 5H,I).

### *Lrp2* KO mice display abnormal epicardial-myocardial development

**Fig. 6.**
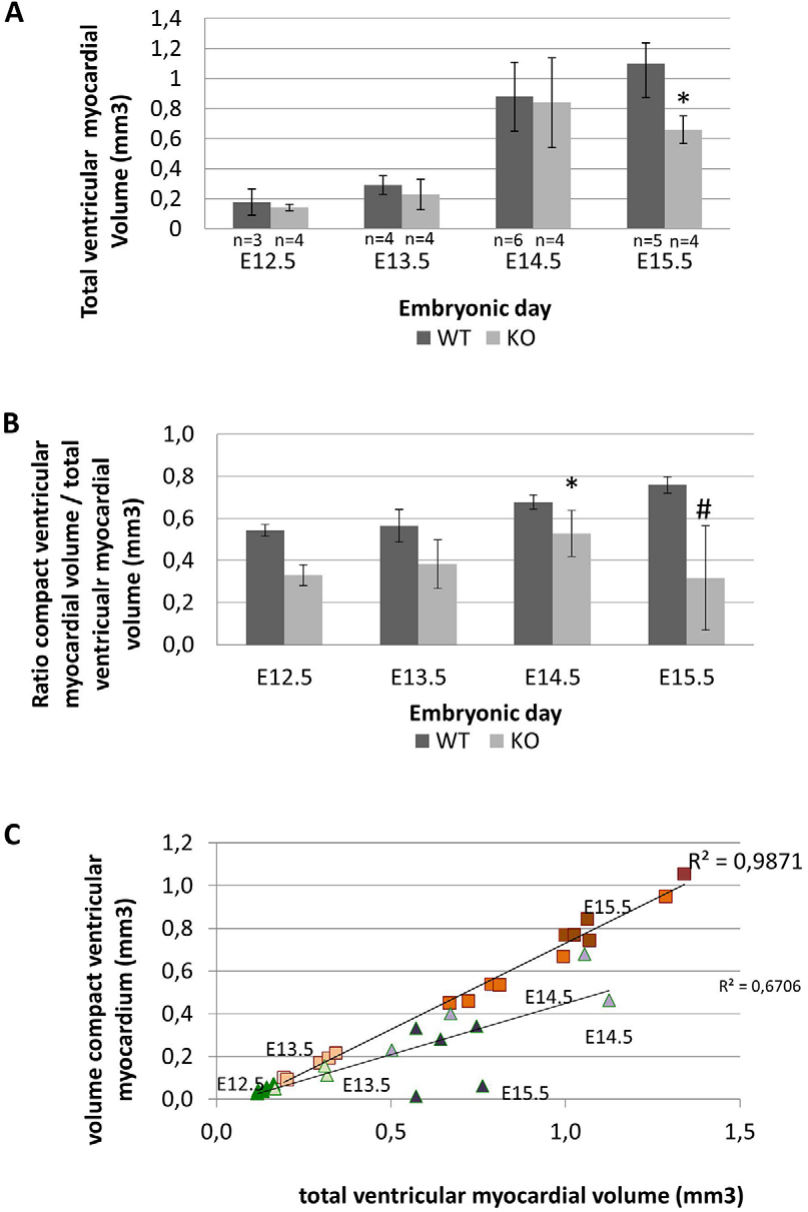
**Myocardial morphometry of the ventricular myocardium between E12.5 and E15.5 in *Lrp2* knockout and wild-type embryos.** (A) The absolute total ventricular myocardial volumes including standard deviations (s.d.) for each embryonic day (*P=0.008). (B) The ratios of compact ventricular myocardial volumes/total ventricular myocardial volumes including s.d. for each embryonic day (*P=0.019; ^#^*P*=0.008). (C) The distribution of the compact myocardial volumes relative to the total ventricular myocardial volumes for each embryonic day. Each color represents a different developmental stage further indicated by the embryonic day. *R*^2^ regression coefficients are shown for both wild-type (WT; represented as squares) and knockout (KO; represented as triangles) embryos (*R*^2^=0.987 and *R*^2^=0.671). The variation in the KO group is due to a broad range of the severity of the non-compaction of the ventricular myocardium at E14.5 and E15.5.

**Fig. 7.**
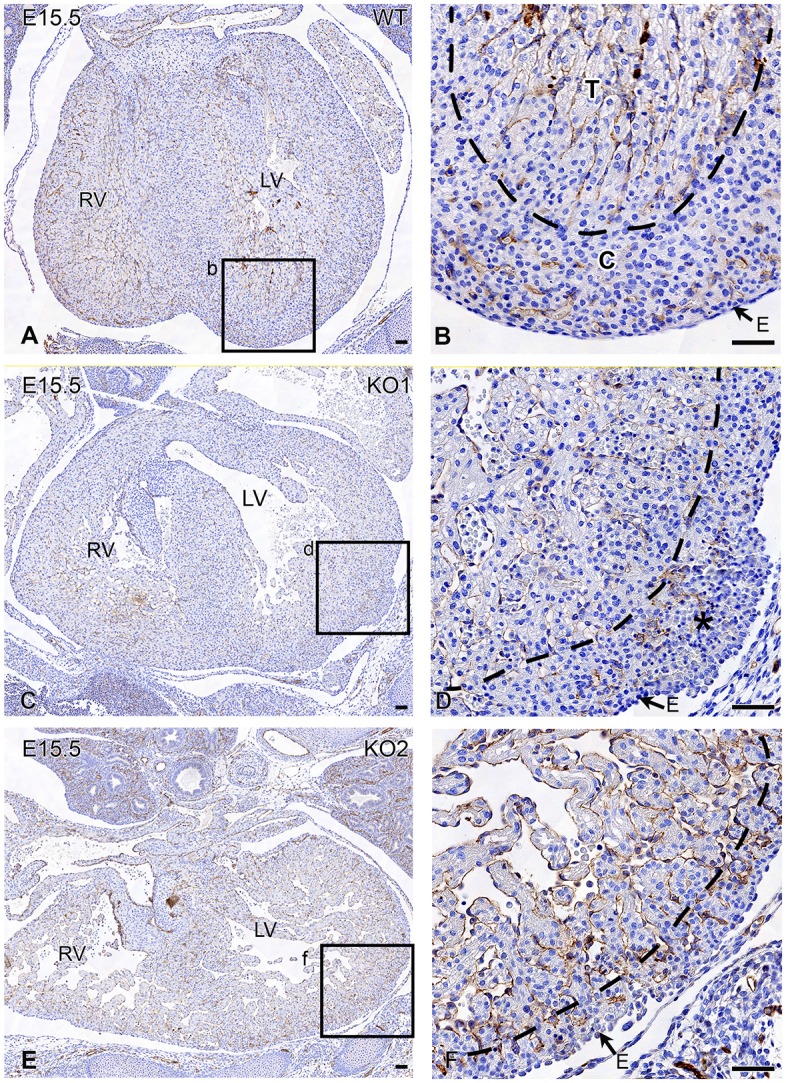
**The coronary endothelium and the endocardium of the left and right ventricle in E15.5 wild-type and two *Lrp2* knockout mice are immunohistochemically stained for CD31 (PECAM).** (A,B) In the wild type (WT), the compact (‘C’ in B) myocardial layer with the coronary vasculature can easily be distinguished (dotted line, B) from the trabecular (T) myocardium. (C,D) Knock out 1 (KO1) is an example with a thick free wall myocardium. Although the compact myocardial thickness of this KO does not seem to be altered considerably at first notice, it contains many abnormal deep intertrabecular spaces. These spaces are connected with the subepicardial vessels (fistulae), resulting in hemorrhages (*). (E,F) KO2 is an example of the severe end of the spectrum, with a very thin walled myocardium. The intertrabecular spaces are clearly visible and have many connections with the subepicardial vessels. E, epicardium; LV, left ventricle; RV, right ventricle. Scale bars: 50 µm.

The total ventricular myocardial wall thickness in E15.5 knockout embryos (*n*=5) versus wild type was significantly reduced (0.66±0.09 and 1.12±0.27 mm^3^, respectively; *P*=0.008; Fig. 6A and compare Fig. 1C with 1D). The thickness of the free wall myocardium varied strongly between the knockout mice (Fig. 7). Of all embryos studied, 14 embryos had abnormal epicardial blebbing and most of them (*n*=13) contained red blood cells on the outer surface of the left ventricle (Table 1, Fig. 7C,D) connecting to deep intertrabecular spaces of the myocardial wall (Fig. 7D,F).

Because *Lrp2* knockout mice display hypoplasia of the ventricular myocardium, we investigated whether this was due to abnormal formation of the compact myocardium. Therefore, we measured the volumes of the compact ventricular myocardial layer including the ventricular septum from E12.5 to E15.5, encompassing the total volume of the ventricular myocardium (compact and trabecular myocardium). In wild-type embryos the total volume of the ventricular myocardium hardly increased from E12.5-E13.5, in contrast to E14.5 and E15.5 where a strong increase is observed (Fig. 6A) corresponding with normal development. Normally, increase of the compact ventricular myocardium is a linear process marked by a regression coefficient that is close to 1 (measured from E12.5-E15.5) (Fig. 6C, *R*^2^ 0.98). The amount of compact myocardium measured as the ratio of the total ventricular myocardium increased strongly at E14.5 and E15.5 (Fig. 6B). This ratio was not significantly reduced in the knockout embryos for E12.5-E13.5, but was significantly reduced for E14.5 and E15.5 (Fig. 6B, *P*=0.019 and *P*=0.008). In mutants we did not observe the same linear process of formation of the compact ventricular myocardium. For the younger stages (E12.5, E13.5 and E14.5) it seems to start with a somewhat linear process; however, at E14.5 and E15.5 (light green and green; Fig. 6C), we observed a lower volume of the compact myocardium relative to the total ventricular myocardium, and wider distributions within each gestation age (*R*^2^ 0.68).

**Fig. 8.**
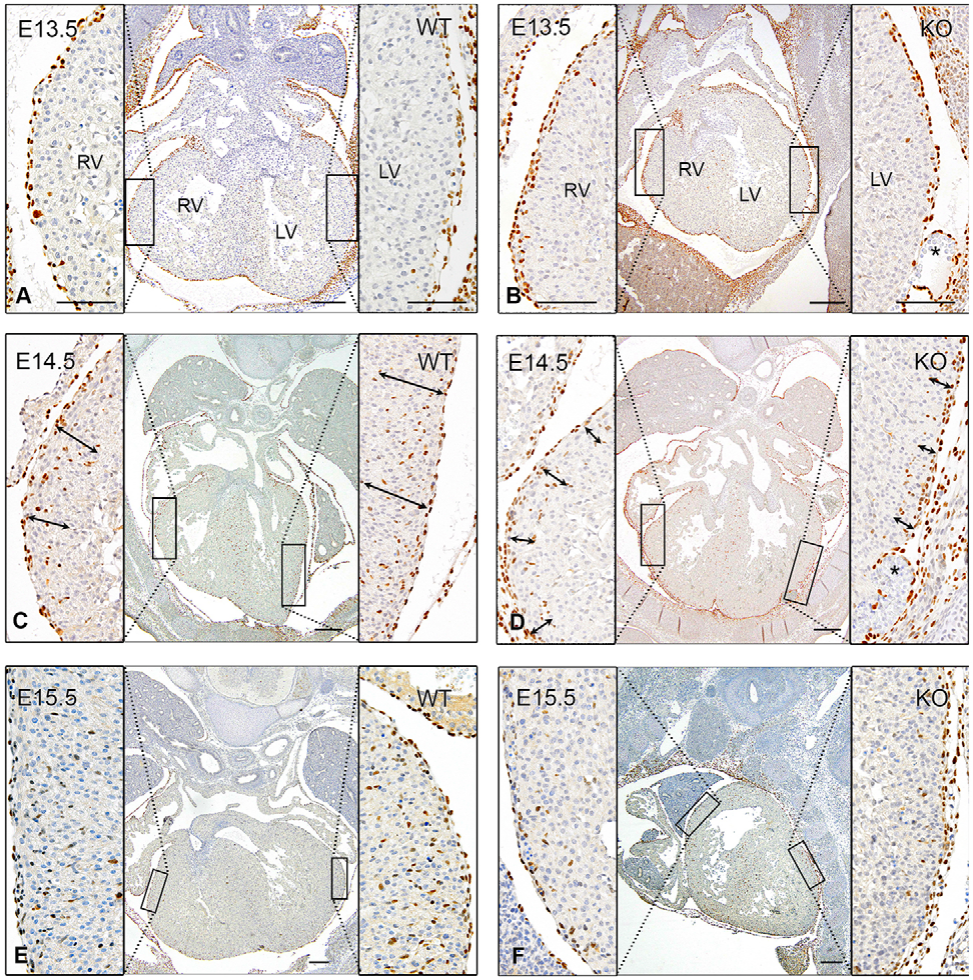
**Epicardial-myocardial changes in *Lrp2* wild-type and knockout mice (E13.5-E15.5) stained with WT1.** At E13.5, the ventricular myocardium is covered with epicardium containing epicardium-derived cells (EPDCs) (A). At E14.5, the EPDCs are found in the compact layer of the ventricular myocardium (C). Myocardial compaction is almost complete at E15.5 (E). In *Lrp2* knockout (KO) embryos, the epicardium is present at E13.5 (B). At many sites, epicardial blebbing with hemorrhage are seen (asterisk). At E14.5, fewer EPDCs are present compared to the wild-type (WT) situation (compare double arrows in WT and KO; C,D). At E15.5, there are hardly any WT1-positive cells located in the myocardium (F). Scale bars: 200 µm (overviews), 50 µm (details). LV, left ventricle; RV, right ventricle.

At E13.5 the epicardium and one to two layers of subepicardial cells were equally present in both wild-type and knockout embryos (Fig. 8A,B). At E14.5 a reduced WT1 expression was observed in the free wall of the right ventricle in the knock out compared to the wild type, the reduction being even more prominent in the free wall of the left ventricle (Fig. 8C,D). At E15.5, WT1-expressing EPDCs were still recognizable in the ventricular myocardial wall; however, the WT1 expression was still more reduced in the knock out (Fig. 8E,F).

## DISCUSSION

LRP2 is crucial for embryonic development in mice and humans and a role has been mentioned in the NCCs (Spoelgen et al., 2005) and in Shh signaling (McCarthy and Argraves, 2003), but a thorough analysis in cardiac development is lacking. Therefore, we investigated the hearts of *Lrp2* knockout embryos in a developmental series between E10.5 and E15.5. For the first time, we were able to demonstrate that LRP2 is crucial for normal cardiac development. Absence of LRP2 resulted in OFT anomalies (CAT, aortic arch and coronary artery anomalies), ventricular septal defects and overriding of the tricuspid valve as well as a marked reduction of the compact layer of the ventricular myocardium, epicardial blebbing and the presence of deep intertrabecular spaces.

**Fig. 9.**
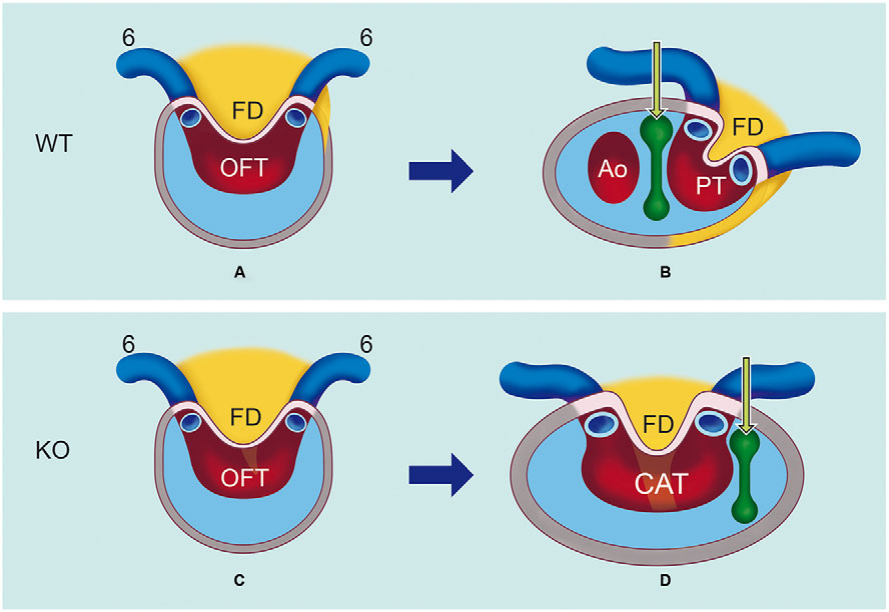
**A schematic depiction of the relative disposition of the SHF and NCCs during OFT septation in *Lrp2* wild-type and knockout mice.** (A) The second heart field (SHF) population (yellow) provides, in both wild-type (WT) and knockout (KO) embryos, a flow divider (FD) in between both 6th pharyngeal arch arteries (dark blue, 6). (B) In WT, the SHF, including the FD, extends and shifts to the left, giving rise to the pulmonary outflow tract (OFT). The extracardiac NCCs (green arrow) migrate at the right side of the SHF towards the OFT cushions and give rise to the aorto-pulmonary septum (green halter) that septates at the orifice level the aortic sac into an ascending aorta (Ao) and a pulmonary trunk (PT). The bridge of the aorto-pulmonary septum also results in septation of the endocardial OFT cushions (pale blue area within the gray myocardium). (C) In the KO embryos, the SHF is also positioned in a mid-sagittal position. However, the outgrowth of the FD is directed in a more anterior direction. (D) During further development the bulk of the SHF population remains in a mid-sagittal position. The right-sided space for the NCCs is therefore not formed. The NCCs are rerouted and take an outward left course, not separating the pulmonary trunk from the aorta. There is extension into the OFT cushions but these do not become fused. This results in a common arterial trunk (CAT) because no proper aorto-pulmonary septum is formed.

Septation of the outflow tract is a process that is orchestrated by the NCCs and the SHF (Anderson et al., 2012; Dyer and Kirby, 2009; Jain et al., 2011; Keyte and Hutson, 2012; Li et al., 2004; Mjaatvedt et al., 2001; Scherptong et al., 2012; Waldo et al., 2001). During lengthening and septation of the OFT, LRP2 is expressed in all the contributing cell populations, i.e. SHF (with the exception of the differentiated myocardium), NCCs, endocardium, epicardium and pericardial mesothelium, indicating the potential for involvement in OFT formation. Here, we show that, in *Lrp2* knockout embryos, OFT septation fails. The SHF population does provide, in both wild-type and knockout embryos, a ridge, which we refer to as an FD, in a mid-sagittal position anterior to the gut that separates in the posterior wall of the aortic sac both 6th pharyngeal arch arteries. In the *Lrp2* knockout embryos the bulk of the SHF population remains in a mid-sagittal position, resulting in absence of the pulmonary push (Scherptong et al., 2012), which is necessary to create a right-sided space in which NCCs can form the aorto-pulmonary septum that septates the aortic sac into a pulmonary and aortic side. Failure of this septation process as seen in CAT in the mutant embryos shows rerouting of the mesenchymal NCC population towards the left (pulmonary side), where these cells connect to the endocardial cushion tissue of the OFT. The pulmonary side of the aortic sac including the connection of the 6th pharyngeal arch arteries and their intermediate FD remain connected to the common OFT. So no NCC-containing aorto-pulmonary septum is formed at the proper side necessary for fusion of the endocardial OFT cushions, resulting in a CAT in 15/17 *Lrp2* knockout embryos. Because our data show that LRP2 is expressed by both the cardiac NCCs and the non-myocardial SHF-derived cells, the combined disturbed remodeling of the NCC and SHF population seems to underlie the OFT anomalies seen in *Lrp2* knockout embryos. Fig. 9 provides a schematic depiction of the relative disposition of the crucial elements for OFT septation.

More than half of the *Lrp2* knockout embryos had an inlet ventricular septal defect (VSD) and overriding tricuspid valve orifice. Also, other studies (e.g. Bartram et al., 2001) have previously demonstrated that OFT abnormalities can coincide with overriding tricuspid valve and inlet VSD, and concluded that this combination of cardiac malformations represents disturbances of the final phase of the cardiac looping process. In our study the defective addition of SHF to the OFT is accompanied by a deficient movement of the atrioventricular canal to the right. This process can be seen as final stage deficient looping and leads to malalignment of the various septal components (Gittenberger-de Groot et al., 2014). In the case of the *Lpr2* mutant, the OFT septation can be appreciated as being completely out of line, whereas primary atrial and inlet septum are malaligned with subsequent overriding of the tricuspid orifice accompanied by an inlet VSD.

Secondly, the process of formation of the compact ventricular myocardium was severely disturbed. This process is orchestrated by EPDCs that migrate from the epicardium around E13.5 into the ventricular myocardium, reviewed by Gittenberger-de Groot et al. (2012), where they contribute to the myocardial architecture as interstitial fibroblasts (Gittenberger-de Groot et al., 1998). Normally, at E15.5 the process of formation of the compact myocardium is almost finished. At E15.5, there was a significant reduction in the volume of the compact myocardium in the knockout embryos. The epicardium showed severe blebbing at several sites with blood-filled spaces that seemed to be in contact with deep ventricular intertrabecular spaces. These anomalies coincided with a reduced WT1 expression. These data suggest that downstream signaling that determines EPDC differentiation into fibroblasts and the necessary interaction of EPDCs with the myocardial cells to form the compact layer of the ventricular myocardium is disturbed, as has been described for other animal models (Gittenberger-de Groot et al., 2012). Also, the observation of a great number of single coronary arteries in the CAT cases of the mutant embryos points towards an epicardial disturbance (Eralp et al., 2005). The focus on a possible primary epicardial problem is based on the observation that Lrp2 is temporarily expressed in the epicardium at E13.5 and in the epicardium around the great arteries at E14.5, but not in the differentiated ventricular myocardium.

### Resemblance of the *Lrp2* knockout with other animal models points towards disturbed RA and Shh signaling

Retinoic acid (RA) is crucial for cardiac development, especially for the OFT and the formation of the compaction ventricular myocardium (Jiang et al., 2002; Niederreither et al., 2001). Because LRP2 is a retinol-binding protein receptor (Christensen et al., 1999) and the *Lrp2* knockout mouse displays both OFT anomalies and a less-compact ventricular myocardium as demonstrated in this manuscript, the underlying mechanism is suspected to be disturbed RA signaling. Other knockout models point in the same direction. Retinoic acid receptor (RAR) knockouts, for example *RARα1/RARβ* double knockouts, have a 100% penetrance of CAT (Jiang et al., 2002). In these mice a marked shortened OFT arises from the right ventricle. RA signaling is carried by the SHF and the cardiac NCCs (Dyer and Kirby, 2009). Interestingly, Jiang et al. showed that, in *RARα1/RARβ* knockout mice, the number, migration and differentiation of NCCs were normal, highly resembling our *Lrp2* knockout mice (Jiang et al., 2002). Furthermore, resemblance of the phenotype of individuals with a *STRA6* mutation (Mathew-Woods syndrome) with individuals with an *LRP2* mutation (Donnai-Barrow syndrome) is obvious, as is the resemblance of a zebrafish model in which STRA6 function is disrupted (Isken et al., 2008; Scherz et al., 2008; Segel et al., 2009) with the Lrp2 knockout mouse. The phenotype in humans with a *STRA6* mutation includes OFT anomalies, whereas extracardiac anomalies highly resemble the phenotype of the *Lrp2* knockout mouse, and humans with an *LRP2* mutation with diaphragmatic hernia and anophthalmia. STRA6 is the most important RBP receptor in humans (Sun, 2012). Unfortunately, little is known about STRA6 expression during heart development, and the cardiac phenotype in the zebrafish model has not been studied in detail as well. Further studies are necessary to elucidate how co-expression of STRA6 with LRP2 affects RBP uptake during OFT formation and myocardial compaction.

It is possible that the multi-ligand receptor LRP2 influences different pathways during cardiac development or maybe even plays an as-yet-unknown key role in connecting pathways, as has been seen for TBX1 (Garg et al., 2001), which influences both Shh- and RA-dependent signaling (Liao et al., 2008). We know that morphogens like Shh and RA act in parallel with other pathways, such as the BMP and Nodal pathway, in the regulation of shared target genes (Maynard et al., 2013; Wong et al., 2012). Future studies should unravel the position of Lrp2 in these networks of genes.

The Sonic hedgehog (SHH)-GLI pathway plays a role in many syndromes associated with septal defects (reviewed by Gittenberger-de Groot et al., 2014). The primary cilium present on the endocardial cells is involved in this signaling process, which controls the endocardial-mesenchymal transformation in the cardiac cushions. *LRP2* has been recently recognized as a cilium-related gene (Li et al., 2015). Our staining for LRP2 expression demonstrated endocardial expression in the OFT cushions but did not reveal a distinct expression in the atrioventricular cushions. Although we cannot rule out a role of LRP2 in the atrioventricular cushions, the described malformations at the inflow seems to be more related to the disturbed looping processes described above than to hampered endothelial-mesenchymal transformation.

### LRP2 as a cholesterol transporter on the yolk sac and placenta as a possible causative factor in the multifactorial model

Cholesterol is important for embryonic development. Individuals with Smith-Lemli-Opitz syndrome, an autosomal-recessive disease of endogenous cholesterol synthesis, display a broad range of congenital anomalies resembling those in individuals with Donnai-Barrow syndrome (Digilio et al., 2003; Smith et al., 1964). The underlying mechanism in Smith-Lemli-Opitz syndrome is a disturbed SHH-GLI signal transduction: cholesterol is essential to activate this pathway (Digilio et al., 2003).

It is postulated that besides endogenous cholesterol synthesis also maternal-fetal cholesterol transport across the yolk sac and placenta is important for embryonic development (Baardman et al., 2013; Woollett, 2005). LRP2 functioning is intriguing because it is expressed at all maternal-fetal interfaces throughout development (Woollett, 2005). Furthermore, ligands capable of binding to LRP2 are essential for embryonic development as mentioned before. The question raised is whether a disturbed maternal-fetal cholesterol transport can systemically cause the anomalies seen in *Lrp2* knockout mice or, alternatively, whether defects in fundamental local signaling cascades such as disturbed Wnt-BMP4 signaling, Shh signaling, RA signaling, or even impaired local uptake of cholesterol by the heart itself are the underlying causes. Targeted disruption of Lrp2 specific for the neural floor plate during embryogenesis resulted in offspring with the same neural tube and forebrain anomalies as seen in the complete knockout, which suggests that the function of LRP2 in signaling cascades is likely causing the neurological phenotype (Hammes et al., 2005; Spoelgen et al., 2005). Impaired NCC development and differentiation plays a key role in the neural tube and forebrain defects in the *Lrp2* knockout mouse, together with disturbed Shh signaling (Hammes et al., 2005). Possible future studies to reveal the exact underlying mechanism could include a yolk-sac- and placenta-specific *Lrp2* knockout in which the heart is thoroughly examined and functional studies conducted that analyze the cholesterol transport function of LRP2 *in utero*.

In summary, *Lrp2* knockout mice display a range of cardiovascular abnormalities resulting from failure of OFT septation, failure of a normal ventricular inlet formation, and failure of formation of the compact ventricular myocardium. The underlying process for OFT abnormalities are severe abnormalities in the distribution of both NCCs and SHF-derived cells, and development towards the base of the OFT. The underlying process for failure of the formation of the compact ventricular myocardium remains unclear but seems to be related to deficient EPDC interaction with the ventricular myocardium. Disturbed downstream signaling in which LRP2 functions in the RA pathway is a potential causative factor. However, we contemplate that the function of LRP2 on cholesterol transport either in the heart itself or at the level of the maternal-fetal interfaces might also play a significant role in cardiac development.

Based on the range of cardiovascular abnormalities found in *Lrp2* knockout mice, together with the existence of a human phenotype (Donnai-Barrow syndrome) that includes cardiac anomalies resembling those seen in the *Lrp2* knockout mouse, we propose that LRP2 also plays an important role in cardiogenesis in humans, thereby regulating the development of multiple cardiac structures such as the OFT and the cardiac septa by orchestrating the migration of both the SHF and the NCCs. Further research must focus on identifying LRP2 mutations in humans with syndromic CHD, including CHD with associated extracardiac anomalies.

## MATERIALS AND METHODS

### Generation of *Lrp2* knockout mice and harvesting of embryos

Animal use was in accordance with the University Medical Center Groningen (UMCG) institutional guidelines. All animals received humane care and all experiments were reviewed and approved by the local Animal Experimental Committee. Heterozygous *Lrp2*^+/−^ mice on a CB56BL/6 background were kindly provided by Thomas Willnow (Max Delbrück Center, Berlin, Germany). *Lrp2*^+/−^ animals were housed in the animal facility under standard conditions. Food and water were provided *ad libitum*. *Lrp2*^+/−^ males were crossed with heterozygous females. We observed from 26 litters a normal mendelian ratio of *Lrp2*^+/+^ (*n*=36), *Lrp2*^+/−^ (*n*=67) and *Lrp2*^−/−^ (*n*=38) embryos. The average number of embryos per pregnancy was 8.2. The day of appearance of the vaginal plug was considered as E0.5. Pregnant females were sacrificed, embryos harvested and the whole embryo (E10.5-E12.5) or solely the thorax (E13.5-E15.5) were isolated.

Per age group we randomly selected wild-type (E12.5: *n*=3, E13.5: *n*=4, E14.5: *n*=6, E15.5: *n*=5) and knockout (E12.5: *n*=4, E13.5: *n*=4, E14.5: *n*=4, E15.5: *n*=5) embryos to phenotype the cardiac malformations. Additionally, we studied the contribution of the NCCs and SHF in E10.5 (*n*=2×4) and E11.5 (*n*=2×4) embryos. Furthermore, we analyzed five *Lrp2*^+/−^ embryos (E15.5) to confirm or refute that heterozygous *Lrp2* mice present with a phenotype (Willnow et al., 1996).

The collected embryos were immediately fixed in 4% paraformaldehyde in phosphate buffer for 24-48 h and routinely processed for paraffin immunohistochemical evaluation. Serial transverse sections with a slice thickness of 5 µm were mounted onto Star frost glass slides (Menzel-Gläzer) in a 1-to-5 order, by which 5 different stainings on subsequent sections could be compared.

### Genotyping

PCR genotyping was performed on embryonic yolk sac DNA or tail DNA using the Extract-N-Amp™ Tissue PCR Kit (Sigma-Aldrich, Zwijndrecht, The Netherlands), following the standard manufacturer protocol. The wild-type allele was amplified by the primer Lrp2-E+T-F (G21)+Lrp2-E-R (G20) (Table S1), producing a 361-bp fragment. Detection of the mutant allele was performed using the primers Lrp2-E+T-F (G21)+Lrp2-T-R (BPA) (Table S1), resulting in a 300-bp product.

### Immunohistochemistry

After deparaffination and rehydration of the slides, immunohistochemical staining was performed with the antibodies anti-atrial myosin light chain 2a (MLC-2a) as a myocardial marker (1/4000, kindly provided by S. W. Kubalak, Medical University of South Carolina, SC), anti-Nkx2.5 as a (pre)myocardial marker and a marker for SHF-derived cells (1/4000, Santa Cruz Biotechnology, Inc., CA, SC-8697), anti-LPR2 (1/800, kindly provided by J. Herz, University of Texas Southwestern Medical Center, TX), anti-WT1 as a marker for the epicardium and early migrating EPDCs (1/500, Calbiochem, ONCOCA1026), anti-Tfap2α as an NCC marker (1/500, GeneTex Inc., GTX62588) and anti-CD31 (M-20) as a marker for endothelium and endocardium (1/4000, Santa Cruz Biotechnology, Inc., CA, SC-1506). The subsequent steps in the immunohistochemical procedure with diaminobenzidine for light microscopy are thoroughly described in a recent paper by our group (Scherptong et al., 2012).

Double-fluorescence immunostaining was performed with the primary antibodies 1/1000 Nkx2.5 and 1/500 Tfap2α to differentiate between cells of the SHF and NCCs. 1/200 horse anti-goat-Biotin in 1/66 normal horse serum was used in the second antibody incubation step, followed by the third incubation using 1/200 Alexa-Fluor^®^-488–streptavidin (Invitrogen, S-11223) and 1/200 Alexa-Fluor^®^-555–donkey anti-rabbit (Invitrogen, A-31572). For LRP2 expression in the OFT of the heart, 1/1000 anti-LRP2 was used with the second antibody Alexa-Fluor^®^-555–donkey anti-rabbit in combination with 1/1000 Nkx2.5 followed by 1/200 horse anti-goat-Biotin in 1/66 normal horse serum and 1/200 Alexa-Fluor^®^-488–streptavidin (Invitrogen, S-11223). LRP2 in combination with 1/400 Tropomyosine (T9283, Sigma-Aldrich Chemie) were used with the second antibody 1/200 Alexa-Fluor^®^-488–donkey anti-mouse (Invitrogen, A-21202). All incubations were in PBS-Tween followed by 3× rinsing in between each staining step. The sections were counterstained with Prolong gold/DAPI (Invitrogen, P36931) and examined with a Leica DMIRE2 microscope with a NUANCE2 multi-spectral imaging system version 2.8.

### Morphological examination and 3D reconstruction

3D reconstructions were generated of representative E10.5, E11.5 and E15.5 wild-type and knockout embryos, displaying the morphological differences. The reconstructions were made as described earlier (Scherptong et al., 2012), establishing the various stainings and the relative position of the NCC and SHF populations in each reconstruction, using the AMIRA software package version 5.4 (Template Graphics Software, San Diego, CA). Reconstructions can be downloaded from: http://www.caskanatomy.info/research/supplement_megalin.html.

### Myocardial morphometry

We performed morphometry on the ventricular myocardium (E12.5-E15.5) and the endocardial cushions (E10.5-E14.5) using the method of Gunderson et al. based on Cavalieri's principle (Gundersen et al., 1988). In short, to measure ventricular myocardium we randomly positioned regularly spaced points (49 mm^2^ grid for E12.5 and E13.5, and 100 mm^2^ for E14.5 and E15.5) on the MLC-2a-stained ventricular myocardium of embryos from E12.5 to E15.5. We compared the total ventricular myocardial volumes of wild-type embryos and *Lrp2* knockout embryos from different litters. For E15.5 we also analyzed five available heterozygous embryos. After analyzing the five heterozygous embryos of E15.5 and confirming that heterozygous *Lrp2* embryos did not have a phenotype, we counted two heterozygous embryos of E12.5 and included them in the wild-type group for this embryonic day. We established the percentage of compact and trabecular myocardium in these wild-type and knockout embryos. The distance between the subsequent sections of the slides was either 0.075 mm or 0.01 mm depending on the number of sections that differed between the embryos. Ten sections were considered to be sufficient for morphometry. Volume measurements were performed using an Olympus microscope with a 40× or 100× magnification. The distance between the subsequent sections of the slides was either 0.025 mm or 0.05 mm.

### Statistics

Statistical analysis on the volume measurement was performed with Mann-Whitney *U*-test because the data were not perfectly normally distributed and because of low sample size. We calculated the regression coefficient for both wild-type and knockout embryos for the ratio of compact myocardium/total ventricular myocardium to analyze the growth of the compact myocardium relative to the growth of the total ventricular myocardium. A regression coefficient close to 1 was considered to be a linear relation between the two. All data of the volume measurements have been presented as average±s.d. in the graphs. Significance was assumed when *P*<0.05 using the SPSS 20.0 software program (SPSS Inc., Chicago, USA).

## References

[DMM022053C1] Anderson R. H., Chaudhry B., Mohun T. J., Bamforth S. D., Hoyland D., Phillips H. M., Webb S., Moorman A. F. M., Brown N. A. and Henderson D. J. (2012). Normal and abnormal development of the intrapericardial arterial trunks in humans and mice. *Cardiovasc. Res.* 95, 108-115. 10.1093/cvr/cvs147 22499773 PMC4228308

[DMM022053C2] Assemat E., Chatelet F., Chandellier J., Commo F., Cases O., Verroust P. and Kozyraki R. (2005). Overlapping expression patterns of the multiligand endocytic receptors cubilin and megalin in the CNS, sensory organs and developing epithelia of the rodent embryo. *Gene Expr. Patterns.* 6, 69-78. 10.1016/j.modgep.2005.04.014 16027047

[DMM022053C3] Baardman M. E., Kerstjens-Frederikse W. S., Berger R. M. F., Bakker M. K., Hofstra R. M. W. and Plosch T. (2013). The role of maternal-fetal cholesterol transport in early fetal life: current insights. *Biol. Reprod.* 88, 24 10.1095/biolreprod.112.102442 23153566

[DMM022053C4] Bajolle F., Zaffran S., Kelly R. G., Hadchouel J., Bonnet D., Brown N. A. and Buckingham M. E. (2006). Rotation of the myocardial wall of the outflow tract is implicated in the normal positioning of the great arteries. *Circ. Res.* 98, 421-428. 10.1161/01.RES.0000202800.85341.6e 16397144

[DMM022053C5] Bartram U., Molin D. G. M., Wisse L. J., Mohamad A., Sanford L. P., Doetschman T., Speer C. P., Poelmann R. E. and Gittenberger-de Groot A. C. (2001). Double-outlet right ventricle and overriding tricuspid valve reflect disturbances of looping, myocardialization, endocardial cushion differentiation, and apoptosis in TGFß2-knockout mice. *Circulation* 103, 2745-2752. 10.1161/01.CIR.103.22.2745 11390347

[DMM022053C6] Christensen E. I. and Verroust P. J. (2002). Megalin and cubilin, role in proximal tubule function and during development. *Pediatr. Nephrol.* 17, 993-999. 10.1007/s00467-002-0956-5 12478347

[DMM022053C7] Christensen E. I., Moskaug J. O., Vorum H., Jacobsen C., Gundersen T. E., Nykjaer A., Blomhoff R., Willnow T. E. and Moestrup S. K. (1999). Evidence for an essential role of megalin in transepithelial transport of retinol. *J. Am. Soc. Nephrol.* 10, 685-695. 10203351 10.1681/ASN.V104685

[DMM022053C8] Digilio M. C., Marino B., Giannotti A., Dallapiccola B. and Opitz J. M. (2003). Specific congenital heart defects in RSH/Smith-Lemli-Opitz syndrome: postulated involvement of the sonic hedgehog pathway in syndromes with postaxial polydactyly or heterotaxia. *Birth Defects Res. A Clin. Mol. Teratol.* 67, 149-153. 10.1002/bdra.10010 12797454

[DMM022053C9] Dyer L. A. and Kirby M. L. (2009). The role of secondary heart field in cardiac development. *Dev. Biol.* 336, 137-144. 10.1016/j.ydbio.2009.10.009 19835857 PMC2794420

[DMM022053C10] Eralp I., Lie-Venema H., DeRuiter M. C., Van Den Akker N. M. S., Bogers A. J. J. C., Mentink M. M. T., Poelmann R. E. and Gittenberger-de Groot A. C. (2005). Coronary artery and orifice development is associated with proper timing of epicardial outgrowth and correlated Fas ligand associated apoptosis patterns. *Circ. Res.* 96, 526-534. 10.1161/01.RES.0000158965.34647.4e 15705966

[DMM022053C11] Fisher C. E. and Howie S. E. M. (2006). The role of megalin (LRP-2/Gp330) during development. *Dev. Biol.* 296, 279-297. 10.1016/j.ydbio.2006.06.007 16828734

[DMM022053C12] Garg V., Yamagishi C., Hu T., Kathiriya I. S., Yamagishi H. and Srivastava D. (2001). Tbx1, a DiGeorge syndrome candidate gene, is regulated by sonic hedgehog during pharyngeal arch development. *Dev. Biol.* 235, 62-73. 10.1006/dbio.2001.0283 11412027

[DMM022053C13] Gittenberger-de Groot A. C., Vrancken Peeters M.-P. F. M., Mentink M. M. T., Gourdie R. G. and Poelmann R. E. (1998). Epicardium-derived cells contribute a novel population to the myocardial wall and the atrioventricular cushions. *Circ. Res.* 82, 1043-1052. 10.1161/01.RES.82.10.1043 9622157

[DMM022053C14] Gittenberger-de Groot A. C., Vrancken Peeters M.-P. F. M., Bergwerff M., Mentink M. M. T. and Poelmann R. E. (2000). Epicardial outgrowth inhibition leads to compensatory mesothelial outflow tract collar and abnormal cardiac septation and coronary formation. *Circ. Res.* 87, 969-971. 10.1161/01.RES.87.11.969 11090540

[DMM022053C15] Gittenberger-de Groot A. C., Winter E. M., Bartelings M. M., Goumans M. J., DeRuiter M. C. and Poelmann R. E. (2012). The arterial and cardiac epicardium in development, disease and repair. *Differentiation* 84, 41-53. 10.1016/j.diff.2012.05.002 22652098

[DMM022053C16] Gittenberger-de Groot A. C., Calkoen E. E., Poelmann R. E., Bartelings M. M. and Jongbloed M. R. M. (2014). Morphogenesis and molecular considerations on congenital cardiac septal defects. *Ann. Med.* 46, 640-652. 10.3109/07853890.2014.959557 25307363

[DMM022053C17] Gueth-Hallonet C., Santa-Maria A., Verroust P. and Maro B. (1994). Gp330 is specifically expressed in outer cells during epithelial differentiation in the preimplantation mouse embryo. *Development* 120, 3289-3299. 7720568 10.1242/dev.120.11.3289

[DMM022053C18] Gundersen H. J. G., Bendtsen T. F., Korbo L., Marcussen N., Moller A., Nielsen K., Nyengaard J. R., Pakkenberg B., Sorensen F. B., Vesterby A. et al. (1988). Some new, simple and efficient stereological methods and their use in pathological research and diagnosis. *APMIS* 96, 379-394. 10.1111/j.1699-0463.1988.tb05320.x 3288247

[DMM022053C19] Hammes A., Andreassen T. K., Spoelgen R., Raila J., Hubner N., Schulz H., Metzger J., Schweigert F. J., Luppa P. B., Nykjaer A. et al. (2005). Role of endocytosis in cellular uptake of sex steroids. *Cell* 122, 751-762. 10.1016/j.cell.2005.06.032 16143106

[DMM022053C20] Isken A., Golczak M., Oberhauser V., Hunzelmann S., Driever W., Imanishi Y., Palczewski K. and von Lintig J. (2008). RBP4 disrupts vitamin A uptake homeostasis in a STRA6-deficient animal model for Matthew-Wood syndrome. *Cell Metab.* 7, 258-268. 10.1016/j.cmet.2008.01.009 18316031 PMC2561276

[DMM022053C21] Jain R., Engleka K. A., Rentschler S. L., Manderfield L. J., Li L., Yuan L. and Epstein J. A. (2011). Cardiac neural crest orchestrates remodeling and functional maturation of mouse semilunar valves. *J. Clin. Invest* 121, 422-430. 10.1172/JCI44244 21157040 PMC3007154

[DMM022053C22] Jiang X., Choudhary B., Merki E., Chien K. R., Maxson R. E. and Sucov H. M. (2002). Normal fate and altered function of the cardiac neural crest cell lineage in retinoic acid receptor mutant embryos. *Mech. Dev.* 117, 115-122. 10.1016/S0925-4773(02)00206-X 12204252

[DMM022053C23] Kantarci S., Al-Gazali L., Hill R. S., Donnai D., Black G. C. M., Bieth E., Chassaing N., Lacombe D., Devriendt K., Teebi A. et al. (2007). Mutations in LRP2, which encodes the multiligand receptor megalin, cause Donnai-Barrow and facio-oculo-acoustico-renal syndromes. *Nat. Genet.* 39, 957-959. 10.1038/ng2063 17632512 PMC2891728

[DMM022053C24] Keyte A. and Hutson M. R. (2012). The neural crest in cardiac congenital anomalies. *Differentiation* 84, 25-40. 10.1016/j.diff.2012.04.005 22595346 PMC3389200

[DMM022053C25] Korenberg J. R., Argraves K. M., Chen X.-N., Tran H., Strickland D. K. and Argraves W. S. (1994). Chromosomal localization of human genes for the LDL receptor family member glycoprotein 330 (LRP2) and its associated protein RAP (LRPAP1). *Genomics* 22, 88-93. 10.1006/geno.1994.1348 7959795

[DMM022053C26] Li S., Zhou D., Lu M. M. and Morrisey E. E. (2004). Advanced cardiac morphogenesis does not require heart tube fusion. *Science* 305, 1619-1622. 10.1126/science.1098674 15361625

[DMM022053C27] Li Y., Klena N. T., Gabriel G. C., Liu X., Kim A. J., Lemke K., Chen Y., Chatterjee B., Devine W., Damerla R. R. et al. (2015). Global genetic analysis in mice unveils central role for cilia in congenital heart disease. *Nature* 521, 520-524. 10.1038/nature14269 25807483 PMC4617540

[DMM022053C28] Liao J., Aggarwal V. S., Nowotschin S., Bondarev A., Lipner S. and Morrow B. E. (2008). Identification of downstream genetic pathways of Tbx1 in the second heart field. *Dev. Biol.* 316, 524-537. 10.1016/j.ydbio.2008.01.037 18328475 PMC2494702

[DMM022053C29] Maynard T. M., Gopalakrishna D., Meechan D. W., Paronett E. M., Newbern J. M. and LaMantia A.-S. (2013). 22q11 Gene dosage establishes an adaptive range for sonic hedgehog and retinoic acid signaling during early development. *Hum. Mol. Genet.* 22, 300-312. 10.1093/hmg/dds429 23077214 PMC3526161

[DMM022053C30] McCarthy R. A. and Argraves W. S. (2003). Megalin and the neurodevelopmental biology of sonic hedgehog and retinol. *J. Cell Sci.* 116, 955-960. 10.1242/jcs.00313 12584240

[DMM022053C31] Mjaatvedt C. H., Nakaoka T., Moreno-Rodriguez R., Norris R. A., Kern M. J., Eisenberg C. A., Turner D. and Markwald R. R. (2001). The outflow tract of the heart is recruited from a novel heart-forming field. *Dev. Biol.* 238, 97-109. 10.1006/dbio.2001.0409 11783996

[DMM022053C32] Moestrup S. K., Birn H., Fischer P. B., Petersen C. M., Verroust P. J., Sim R. B., Christensen E. I. and Nexo E. (1996). Megalin-mediated endocytosis of transcobalamin-vitamin-B12 complexes suggests a role of the receptor in vitamin-B12 homeostasis. *Proc. Natl. Acad. Sci. USA* 93, 8612-8617. 10.1073/pnas.93.16.8612 8710919 PMC38721

[DMM022053C33] Moore A. W., McInnes L., Kreidberg J., Hastie N. D. and Schedl A. (1999). YAC complementation shows a requirement for Wt1 in the development of epicardium, adrenal gland and throughout nephrogenesis. *Development* 126, 1845-1857. 10101119 10.1242/dev.126.9.1845

[DMM022053C34] Niederreither K., Vermot J., Messaddeq N., Schuhbaur B., Chambon P. and Dolle P. (2001). Embryonic retinoic acid synthesis is essential for heart morphogenesis in the mouse. *Development* 128, 1019-1031. 11245568 10.1242/dev.128.7.1019

[DMM022053C35] Pober B. R., Longoni M. and Noonan K. M. (2009). A review of Donnai-Barrow and facio-oculo-acoustico-renal (DB/FOAR) syndrome: clinical features and differential diagnosis. *Birth Defects Res. A Clin. Mol. Teratol.* 85, 76-81. 10.1002/bdra.20534 19089858 PMC2882234

[DMM022053C36] Poelmann R. E., Mikawa T. and Gittenberger-de Groot A. C. (1998). Neural crest cells in outflow tract septation of the embryonic chicken heart: differentiation and apoptosis. *Dev. Dyn.* 212, 373-384. 10.1002/(SICI)1097-0177(199807)212:3<373::AID-AJA5>3.0.CO;2-E 9671941

[DMM022053C37] Scherptong R. W. C., Jongbloed M. R. M., Wisse L. J., Vicente-Steijn R., Bartelings M. M., Poelmann R. E., Schalij M. J. and Gittenberger-de Groot A. C. (2012). Morphogenesis of outflow tract rotation during cardiac development: the pulmonary push concept. *Dev. Dyn.* 241, 1413-1422. 10.1002/dvdy.23833 22826212

[DMM022053C38] Scherz P. J., Huisken J., Sahai-Hernandez P. and Stainier D. Y. R. (2008). High-speed imaging of developing heart valves reveals interplay of morphogenesis and function. *Development* 135, 1179-1187. 10.1242/dev.010694 18272595

[DMM022053C39] Segel R., Levy-Lahad E., Pasutto F., Picard E., Rauch A., Alterescu G. and Schimmel M. S. (2009). Pulmonary hypoplasia-diaphragmatic hernia-anophthalmia-cardiac defect (PDAC) syndrome due to STRA6 mutations--what are the minimal criteria? *Am. J. Med. Genet. A* 149A, 2457-2463. 10.1002/ajmg.a.33038 19839040

[DMM022053C40] Smith D. W., Lemli L. and Opitz J. M. (1964). A newly recognized syndrome of multiple congenital anomalies. *J. Pediatr.* 64, 210-217. 10.1016/S0022-3476(64)80264-X 14119520

[DMM022053C41] Spoelgen R., Hammes A., Anzenberger U., Zechner D., Andersen O. M., Jerchow B. and Willnow T. E. (2005). LRP2/megalin is required for patterning of the ventral telencephalon. *Development* 132, 405-414. 10.1242/dev.01580 15623804

[DMM022053C42] Sun H. (2012). Membrane receptors and transporters involved in the function and transport of vitamin A and its derivatives. *Biochim. Biophys. Acta* 1821, 99-112. 10.1016/j.bbalip.2011.06.010 21704730 PMC3222080

[DMM022053C43] Waldo K., Kumiski D. H., Wallis K. T., Stadt H. A., Hutson M. R., Platt D. H. and Kirby M. L. (2001). Conotruncal myocardium arises from a secondary heart field. *Development* 128, 3179-3188. 11688566 10.1242/dev.128.16.3179

[DMM022053C44] Waldo K. L., Hutson M. R., Stadt H. A., Zdanowicz M., Zdanowicz J. and Kirby M. L. (2005). Cardiac neural crest is necessary for normal addition of the myocardium to the arterial pole from the secondary heart field. *Dev. Biol.* 281, 66-77. 10.1016/j.ydbio.2005.02.011 15848389

[DMM022053C45] Wessels A., van den Hoff M. J. B., Adamo R. F., Phelps A. L., Lockhart M. M., Sauls K., Briggs L. E., Norris R. A., van Wijk B., Perez-Pomares J. M. et al. (2012). Epicardially derived fibroblasts preferentially contribute to the parietal leaflets of the atrioventricular valves in the murine heart. *Dev. Biol.* 366, 111-124. 10.1016/j.ydbio.2012.04.020 22546693 PMC3358438

[DMM022053C46] Willnow T. E., Hilpert J., Armstrong S. A., Rohlmann A., Hammer R. E., Burns D. K. and Herz J. (1996). Defective forebrain development in mice lacking gp330/megalin. *Proc. Natl. Acad. Sci. USA* 93, 8460-8464. 10.1073/pnas.93.16.8460 8710893 PMC38693

[DMM022053C47] Wong K. S., Rehn K., Palencia-Desai S., Kohli V., Hunter W., Uhl J. D., Rost M. S. and Sumanas S. (2012). Hedgehog signaling is required for differentiation of endocardial progenitors in zebrafish. *Dev. Biol.* 361, 377-391. 10.1016/j.ydbio.2011.11.004 22119054

[DMM022053C48] Woollett L. A. (2005). Maternal cholesterol in fetal development: transport of cholesterol from the maternal to the fetal circulation. *Am. J. Clin. Nutr.* 82, 1155-1161. 16332646 10.1093/ajcn/82.6.1155

